# Nocturia Is Associated with Slipping and Falling

**DOI:** 10.1371/journal.pone.0169690

**Published:** 2017-01-06

**Authors:** So Young Kim, Woojin Bang, Min-Su Kim, Bumjung Park, Jin-Hwan Kim, Hyo Geun Choi

**Affiliations:** 1 Department of Otorhinolaryngology-Head & Neck Surgery and Cancer Research Institute, Seoul National University College of Medicine, Seoul, Korea; 2 Department of Urology, Hallym University Sacred Heart Hospital, Hallym University Sacred Heart Hospital, Anyang, Korea; 3 Department of Otorhinolaryngology-Head & Neck Surgery, Korea University Ansan Hospital, Ansan, Korea; 4 Department of Otorhinolaryngology-Head & Neck Surgery, Hallym University College of Medicine, Anyang, Korea; 5 Department of Otorhinolaryngology-Head & Neck Surgery, Hallym University College of Medicine, Seoul, Korea; San Francisco Coordinating Center, UNITED STATES

## Abstract

Several reports have demonstrated associations between falls and nocturia in the elderly. However, little information is available regarding other age groups. This study evaluated the relationship between the frequency of nocturia and falls in men using a large, population-based survey in Korea, and the results were adjusted for various confounding factors. Data from a 2011 Korean community health survey (KCHS) were retrieved for 92,660 men aged 19 to 103 years. Information regarding the history of slips or falls in the past year was collected. The frequency of nocturia was classified as 0, 1, 2, 3, 4, and ≥ 5 instances a night. Walking during the day, education, income, body mass index (BMI), smoking, alcohol consumption, sleep time, stress level and medical histories of hypertension, diabetes mellitus, hyperlipidemia, cerebral stroke, angina or myocardial infarction, arthritis, and osteoporosis were adjusted using multiple logistic regression analysis with complex sampling. A subgroup analysis was conducted for young (19–30 years), middle-aged (31–60 years), and elderly individuals (61+ years). Approximately 14.6% of the men had a history of falls. Their mean age was 42.9 years, which was significantly higher than that of the non-faller group (P < 0.001). An increased frequency of nocturia was associated with increased adjusted odds ratio (AOR) for falls (AOR for 1 instance of nocturia/night = 1.41 [95% confidence interval, 1.33–1.50]; AOR for 2 instances = 1.41 [1.33–1.50]; AOR for 3 instances = 2.00 [1.75–2.28]; AOR for 4 instances = 2.12 [1.73–2.61]; AOR for ≥ 5 instances = 2.02 [1.74–2.36], P < 0.001). In the subgroup analysis, the AORs for falls significantly increased in all age groups as the frequency of nocturia increased.

## Introduction

Falling is a frequent accident that impairs physical performance and health-related quality of life [1–3]. Because elderly individuals face an increased risk of falls, many studies of falls have focused on this population [1–4]. However, young adults and adolescents also experience fall injuries; our previous studies show that approximately 16.8% of the adult population and 13.0% of the adolescent population experiences fall injuries [5]. Thus, the prevalence of and factors related to falls need to be considered in a wide-ranging population.

Several factors associated with falls have been suggested [6], some of which are modifiable. Environmental factors include home hazards, and individual characteristics include fear of falling, sedentary life-style, and inadequate physical training. These are only some of the examples of modifiable fall factors [7–10]. In our previous studies, inadequate sleep and an obese or underweight body mass index (BMI) were also linked to falls [11]. In contrast, the intrinsic functional and physical health status generally reflects the individual’s age, and muscle weakening, cognitive abilities, visual problems, and medical comorbidities are hard to improve [12]. Additionally, some medical disorders, such as metabolic syndrome, osteoporosis, and lower urinary symptoms (LUTS), could be categorized as non-modifiable factors of falls [6,8,13]. Although these fall-related medical disorders require specific management, more attention to and prevention of falls is required. Consequently, it is important to delineate fall-related medical comorbidities in addition to modifiable factors.

Nocturia is a prevalent LUTS that occurs more often as people age [14]. Previous studies have estimated the prevalence of nocturia approximately 49% in 15 or more than 15 years and 69% in 40 or more than 40 years [14–16]. Several reports have demonstrated significant associations between falls and nocturia [13,17,18]. However, most of these studies focused on elderly subjects and considered fracture injuries and subsequent mortality [13,17,18]. We hypothesized that the nocturia is associated with fall down with dose-response manner in the Korean adult men. Thus, the present study evaluated the relationship between the frequency of nocturia and falls using a large, representative population-based study in Korea. Moreover, we investigated how the prevalence of falls correlated with the frequency of nocturia. This study included a wide range of age groups, from young adults to elderly adults. Furthermore, because numerous factors including age, walking during the day, education, income, BMI, smoking, alcohol, sleep time, stress, and medical histories of hypertension, diabetes mellitus, hyperlipidemia, cerebral stroke, angina or myocardial infarction, arthritis, and osteoporosis might be related with both nocturia and fall down, we adjusted these variables using multiple logistic regression analysis.

## Materials and Methods

### Study Population and Data Collection

The Korean community health survey (KCHS) was approved by the Institutional Review Board of the Korea Centers for Disease Control and Prevention (IRB No. 2011-05CON-04-C). Written informed consent was obtained from all the participants prior to the survey. We used KCHS data upon the permission of the Korea Centers for Disease Control and Prevention.

This was a cross-sectional study using data from the KCHS. Data from the KCHS conducted in 2011 were analyzed. The data were collected by the Centers for Disease Control and Prevention of Korea. The survey gathers information through face-to-face, paper-assisted personal interviews between trained interviewers and respondents. The average sample size for the KCHS was 900 subjects in each 253 community units, including 16 metropolitan cities and provinces. Thus, a total of 227,700 (900 participants x 253 units) conducted the KCHS survey. Among these participants, the 103,017 subjects with 19 to 103 years old male were enrolled in the present study. The KCHS used a two-stage sampling process. The first stage selected a sample area (district/town/village) as a primary sample unit according to the number of households in the area, using a probability proportional to the sampling method. In the second stage, the number of households in the selected sample district/town/village were identified to create a household directory. Sample households were selected using systematic sampling methods. This process was used to ensure that the sample units were representative of the entire population [19]. To ensure that the sample was statistically representative of the population, the data collected from the survey was weighted by statisticians based on the sample design [20].

**Fig 1:**
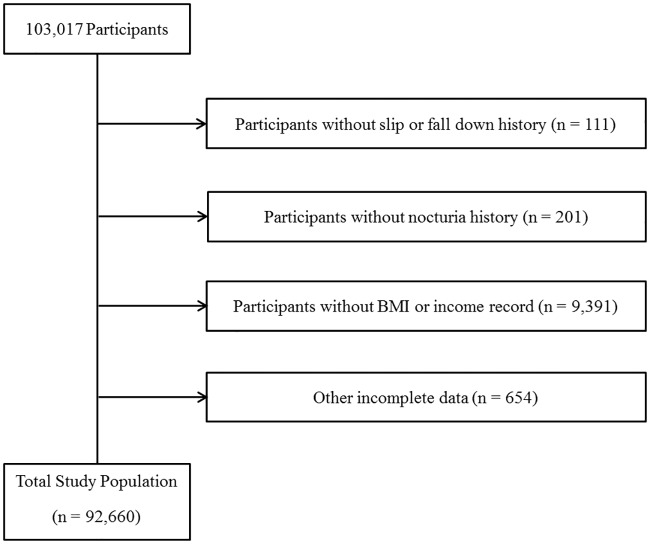
A schematic illustration of the participant selection for the present study. Among a total of 103,017 participants, the participants without a history of fall down (n = 111) or nocturia (201), BMI or income records (9,391) and other incomplete data (654) were excluded. The data for the 92,660 participants from whom complete data were obtained were analysed.

Of a total of 103,017 male participants ranging in age from 19 to 103 years old, we excluded the following groups of participants from this study: those with no records of slipping or falling (111 participants); those who did not fill out the nocturia survey (201 participants); those without height, weight (6,378 participants), or income records (3,013 participants); and those who had incomplete data about education level, smoking, alcohol consumption history, sleep hours, stress level, hypertension, diabetes mellitus, hyperlipidemia, cerebral stroke, angina or myocardial infarction, arthritis, and osteoporosis history (654 participants). Overall, 92,660 participants were included in this study (Fig 1).

### Survey

To measure physical activity, the participants were asked how many days they walked for more than 10 minutes in the past week. To explore the influence of education, uneducated participants and those who had graduated only from elementary or middle school were assigned to the “low” education group; high school graduates comprised the “middle” group; and junior college, graduate school and college graduates formed the “high” group. Participants shorter than 110 cm or weighing less than 30 kg were excluded from this study (1,217 participants). Using the criteria for the Asia-Pacific region [21], three BMI (kg/m^2^) groups were devised: low BMI, < 18.5 kg/m^2^; normal BMI, 18.5–25 kg/m^2^; and high BMI, ≥ 25 kg/m^2^. Using the methods recommended by the Organization for Economic Cooperation and Development [22] (i.e., dividing household income by the square root of the number of household members), monthly income was divided into low, low-middle, upper-middle, and high quartiles. Smoking status was divided into 3 groups: non-smokers, past smokers, and current smokers. Past smokers who had quit smoking less than 1 year previously were included in the current smoker group. Alcohol consumption was divided into the following four categories: None, ≤ 1 instance a month, 2–4 instances a month, and ≥ 2 instances a week. The amount of sleep was divided into three groups: ≤ 6 h per day, 7–8 h per day, and ≥ 9 h per day. Participants who slept less than 3 h per day were excluded from this study (271 participants). The participants were asked if they usually felt stress, and the stress level was divided into the following four groups: no stress, some stress, moderate stress, and severe stress.

The participants were asked about their history of other comorbidities, such as hypertension, diabetes mellitus, hyperlipidemia, cerebral stroke, angina or myocardial infarction, arthritis, and osteoporosis history. Those who reported a history of any of these diseases, as diagnosed by a medical doctor, were recorded as positive.

The participants were asked “How many instances did you typically get up at night to urinate in the past month?” The frequency of nocturia was reported as 0, 1, 2, 3, 4, or ≥ 5 instances per night. The participants were asked “Did you slip or fall down in the past year?” The participants who had a history of slipping or falling were assigned to the slip or fall group. This study included only males because the KCHS only collected nocturia data from male participants.

### Statistical Analysis

The differences in the mean age and walking days of non-faller participants (non-faller) and that of the slip or fall group were compared using linear regression analysis with complex sampling. The differences in marriage, education level, occupation, income level, BMI group, smoking status, alcohol consumption history, sleep hours, stress level, hypertension, diabetes mellitus, hyperlipidemia, and cerebral stroke history were compared using a chi-square test with Rao-Scott correction.

Associations between slipping or falling (yes or no) and nocturia (0 through ≥ 5 instances) were analyzed with various models, including simple logistic regression analysis with complex sampling (unadjusted); multiple logistic regression analysis with complex sampling adjusted for age (model 1); and multiple logistic regression analysis with complex sampling adjusted for age, education, income level, BMI group, smoking, alcohol consumption, stress level, hypertension, diabetes mellitus, cerebral stroke, angina or myocardial infarction, arthritis, and osteoporosis history (model 2). Subgroup analyses were conducted according to age groups (19–30 years old, 31–60 years old, and 61+ years old) using model 2.

Two-tailed analyses were conducted, and *P*-values lower than 0.05 were considered significant. The adjusted odds ratio (AOR) and 95% confidence interval (CI) for falling down were calculated. All results are presented as weighted values. The results were analyzed statistically using SPSS ver. 21.0 (IBM, Armonk, NY, USA).

## Results

Overall, 14.6% of the participants had a history of slipping or falling. The frequency of nocturia within the fall group was significantly different from that of the non-faller group (P < 0.001). All of the retrieved variables showed significant differences between the fall and non-faller groups, which warranted adjustments for the relationship between falls and nocturia. The average age of the fall group was 42.9 years old, which was significantly lower than that of the non-faller group (44.6 years old; P < 0.001). In addition to age, the number of walking days per week, education level, income level, BMI, smoking status, alcohol use, sleep time, stress level, and medical histories of diabetes mellitus, cerebral stroke, angina or myocardial infarction, arthritis, and osteoporosis were significantly different between the fall and non-faller groups according to a chi-square test (P < 0.001 for each except for diabetes mellitus [P = 0.002]; Table 1).

**Table 1:** General characteristic of participants.

	Non-faller	Slip or Fall down	P-value
Number			
N	79,112	13,548	
%	85.4	14.6	
Age (year)	44.6	42.9	<0.001^a^
Walking day (d)	4.2	4.4	<0.001^a^
Education (%)			0.001^b^
Low	16.2	17.5	
Middle	32.3	30.9	
High	51.5	51.6	
Income (%)			<0.001^b^
Lowest	11.5	14.8	
Low-middle	24.3	25.4	
Upper-middle	30.1	28.7	
Highest	34.2	31.1	
BMI (%)			<0.001^b^
<18.5 kg/m^2^	2.3	2.9	
≥18.5 kg/m^2^, <25 kg/m^2^	68.3	66.2	
≥25 kg/m^2^	29.5	30.9	
Smoking (%)			<0.001^b^
None	26.1	24.9	
Past smoker	28.6	27.8	
Current smoker	45.3	47.3	
Alcohol (%)			<0.001^b^
None	15.4	13.3	
≤ 1 instance a month	18.1	17.9	
2–4 instances a month	29.1	29.6	
≥ 2 instances a week	37.4	39.2	
Sleep (%)			<0.001^b^
≤ 6 h	50.2	46.1	
7–8 h	47.1	50.1	
≥ 9 h	2.7	3.8	
Stress (%)			<0.001^b^
No	16.8	16.1	
Some	55.4	50.3	
Moderate	24.4	28.3	
Severe	3.3	5.4	
Hypertension (%)			0.372
Yes	16.9	16.5	
No	83.1	83.5	
Diabetes mellitus (%)			0.002
Yes	6.6	7.4	
No	93.4	92.6	
Hyperlipidemia (%)			0.227
Yes	9.2	9.6	
No	90.8	90.4	
Cerebral Stroke (%)			<0.001^b^
Yes	1.2	2.3	
No	98.8	97.7	
Angina or Myocardial infarction			<0.001^b^
Yes	2.0	2.6	
No	98.0	97.4	
Arthritis (%)			<0.001^b^
Yes	3.2	5.5	
No	96.8	94.5	
Osteoporosis			
Yes	0.6	1.2	
No	99.4	98.8	
Nocturia			<0.001^b^
None	66.9	60.1	
1 instance	21.9	24.4	
2 instances	6.6	8.5	
3 instances	2.3	3.4	
4 instances	0.8	1.3	
≥ 5 instances	1.5	2.4	

^a^ Linear regression analysis with complex sampling, Significance at P < 0.05

^b^ Chi-square test with Rao-Scott correction, Significance at P < 0.05

Because nocturia most frequently occurred 0 to 4 instances per night, the prevalence of falls increased in a dose-dependent manner (OR of 1 instance of nocturia a night = 1.24 [95% CI, 1.17–1.31]; OR of 2 instances = 1.44 [1.33–1.56]; OR of 3 instances = 1.66 [1.47–1.87]; OR of 4 instances = 1.77 [1.46–2.13]; P < 0.001). In cases of ≥ 5 instances of nocturia per night, the OR of falls (OR = 1.72, 95% CI = 1.49–1.98, P < 0.001) was slightly lower compared with that for 4 instances of nocturia per night, although it was still higher than the OR for 3 instances. The relationship between the frequency of nocturia and falls was maintained after adjustment for age (model 1; AOR of 1 instance of nocturia per night = 1.43 [95% CI, 1.35–1.51]; AOR of 2 instances = 1.88 [1.73–2.05]; AOR of 3 instances = 2.36 [2.08–2.67]; AOR of 4 instances = 2.59 [2.13–3.15]; AOR of ≥ 5 instances = 2.34 [2.02–2.71]; P < 0.001) and other variables (model 2; AOR of 1 instance of nocturia per night = 1.41 [95% CI, 1.33–1.50]; AOR of 2 instances = 1.41 [1.33–1.50]; AOR of 3 instances = 2.00 [1.75–2.28]; AOR of 4 instances = 2.12 [1.73–2.61]; AOR of ≥ 5 instances = 2.02 [1.74–2.36]; P < 0.001). The AORs were slightly higher than the ORs in the unadjusted model (Table 2).

**Table 2:** Odd ratios of nocturia (0–5 or more instances) for falling down using multiple logistic regression analysis with complex sampling.

	OR	95% CI	P-value
Unadjusted			<0.001^a^
None	1		
1 instance	1.24	1.17–1.31	
2 instances	1.44	1.33–1.56	
3 instances	1.66	1.47–1.87	
4 instances	1.77	1.46–2.13	
≥ 5 instances	1.72	1.49–1.98	
Model 1^b^			<0.001^a^
None	1		
1 instance	1.43	1.35–1.51	
2 instances	1.88	1.73–2.05	
3 instances	2.36	2.08–2.67	
4 instances	2.59	2.13–3.15	
≥ 5 instances	2.34	2.02–2.71	
Model 2^c^			<0.001^a^
None	1		
1 instance	1.41	1.33–1.50	
2 instances	1.71	1.57–1.87	
3 instances	2.00	1.75–2.28	
4 instances	2.12	1.73–2.61	
≥ 5 instances	2.02	1.74–2.36	

^a^ Significance at P < 0.05

^b^ Adjusted with age

^c^ Adjusted with age, education, income level, BMI group, smoking, alcohol consumption, stress level, hypertension, diabetes mellitus, cerebral stroke, angina or myocardial infarction, arthritis, and osteoporosis history

Subgroup analysis demonstrated a significant positive relationship between nocturia and falls in each age group, which was similar to the relationship for the total adult population. The elderly adults showed a clear positive, dose-dependent relationship between nocturia and falls (AOR of 1 instance of nocturia per night = 1.26 [95% CI, 1.12–1.41]; AOR of 2 instances = 1.36 [1.20–1.54]; AOR of 3 instances = 1.34 [1.15–1.56]; AOR of 4 instances = 1.59 [1.29–1.95]; AOR of ≥ 5 instances = 1.73 [1.41–2.11]; P < 0.001). In the young adults, nocturia was related to falling except in the group with 3 instances of nocturia per night (AOR of 1 instance of nocturia per night = 1.59 [95% CI, 1.38–1.83]; AOR of 2 instances = 1.63 [1.21–2.20]; AOR of 3 instances = 1.50 [0.91–2.50]; AOR of 4 instances = 3.02 [1.06–8.61] AOR of ≥ 5 instances = 3.10 [1.78–5.41]; P <0.001). In the middle-aged group, nocturia was related to falling except in the 4 instances per night group (AOR of 1 instance of nocturia per night = 1.40 [95% CI, 1.30–1.83]; AOR of 2 instances = 1.68 [1.21–2.20]; AOR of 3 instances = 2.35 [1.06–8.61]; AOR of 4 instances = 1.55 [0.94–1.98]; AOR of ≥ 5 instances = 1.53 [1.18–1.98]; P < 0.001;Table 3).

**Table 3:** Subgroup analysis of adjusted odd ratios of nocturia for falling down using multiple logistic regression analysis with complex (Model 2) sampling according to age groups.

Fall down	AOR	95% CI	P-value
Young adults (19–30 years old)			<0.001^a^
None	1		
1 instance	1.59	1.38–1.83	
2 instances	1.63	1.21–2.20	
3 instances	1.50	0.91–2.50	
4 instances	3.02	1.06–8.61	
≥ 5 instances	3.10	1.78–5.41	
Middle age (31–60 years old)			<0.001^a^
None	1		
1 instance	1.40	1.30–1.50	
2 instances	1.68	1.47–1.91	
3 instances	2.35	1.87–2.96	
4 instances	1.55	0.94–2.56	
≥ 5 instances	1.53	1.18–1.98	
Elderly (61+ years old)			<0.001^a^
None	1		
1 instance	1.26	1.12–1.41	
2 instances	1.36	1.20–1.54	
3 instances	1.34	1.15–1.56	
4 instances	1.59	1.29–1.95	
≥ 5 instances	1.73	1.41–2.11	

^a^ Significance at P < 0.05

## Discussion

The prevalence of slipping or falling was 14.6% in the current study. Nocturia was significantly correlated with falls, even after adjusting for other variables. Furthermore, the frequency of nocturia was significantly associated with an elevated AOR of falls until the voiding frequency reached 4 instances a night. The present study is the first to demonstrate the dose response relations between the frequencies of nocturia and falls in adult men.

In line with our results, published studies have demonstrated that LUTS, including nocturia and urinary incontinence, are related to falls in elderly men [17,23,24]. Although definite dose-dependent relationships were not delineated in the present study, a cohort study of elderly individuals demonstrated the relationship of nocturia with falls [17]. In that cohort study, having at least two nocturia instances per night was significantly associated with falls (OR = 1.84, 95% CI = 1.05–3.22), and > 3 instances of voiding per night was associated with a greater OR for falls (OR = 2.15, 95% CI = 1.04–4.44) [17]. Similarly, another prospective cohort study of older subjects also demonstrated a positive relationship between urinary incontinence and falls (OR = 1.8, 95% CI = 1.4–2.4) [23].

However, some studies reported no significant correlation between nocturia and falls [25,26]. Differences in the definitions of nocturia, the types of falls, the size and characteristics of study populations, and possible confounding factors might contribute to these discrepancies. For example, a previous study that demonstrated an insignificant correlation between nocturia and falls defined nocturia as ≥ 3 instances a night [26]. Another study with an insignificant association between nocturia and falls counted only falls that resulted in fracture injuries [25]. The inclusion of a limited number of variables in the logistic regression and a relatively small number of study groups are other possible reasons for an insignificant relationship between nocturia and falls [26]. The present study encompassed numerous possible confounders using multiple logistic regression analysis, which makes this study fills in the gaps left by previous studies. Additionally, the present study included individuals with a wide range of ages, whereas only older individuals were considered in the previous studies [17,23]. Subgroup analysis according to age group demonstrated significant associations between nocturia and falls for all adult age groups and not just the elderly (Table 3).

Furthermore, the frequency of nocturia had a positive dose-response relationship with falls until the frequency of nocturia reached 4 instances per night. The AORs for falls in the young adult and elderly groups showed positive dose-dependent increases from 1 through ≥ 5 instances of nocturia a day. Although nocturia 4 instances per night did not have a significant relationship with falls, the middle-aged group also demonstrated a positive relationship between nocturia and falls, which peaked at 3 instances of nocturia per night. The group reporting ≥ 5 instances of nocturia per night showed AORs for falls slightly lower than those of the other nocturia groups but still higher than those of the non-faller s; Because of their frequent voiding at night, these subjects may have little time to sleep or may sleep with the lights on, resulting in relatively limited opportunities for falls to occur [27].

Nocturia could cause falls at night because several instances of abruptly waking up to void may elevate the risk of incidental falls. In addition, nocturia is known to be a leading cause of sleep fragmentation in elderly individuals [28]. This disturbance or fragmentation of sleep may induce daytime sleepiness, which increases the risk of falls [29]. In addition, the chronic impacts of nocturnal waking due to frequent voiding may result in impaired attention, psychiatric problems and organic diseases [30,31]. It has been reported that the first nocturia episode often occurs at a mean of 2–3 h after sleep begins, during slow-wave sleep [13]. Studies have suggested that waking during slow-wave sleep is detrimental to a positive mood and is related to medical disorders, such as uncontrolled glucose levels in diabetes mellitus, compared with comparable total sleep times [32,33]. These adverse impacts of nocturia on physical and psychological health might mediate the increased prevalence of falls in subjects with nocturia.

In addition to the sleep fragmentation caused by nocturnal voiding, nocturia itself has been suggested to be related to somatic disorders, such as cardiac diseases, uncontrolled diabetes mellitus, and obstructive sleep apnea; mental health disorders, including depression; and impaired quality of life, which are associated with the increased incidence of falling [34–37]. Inversely, the fall groups showed higher levels of several comorbidities that could cause nocturia. The pathophysiologic mechanisms of nocturia are complicated [38]. Several anatomic causes, primarily including benign prostatic obstruction and metabolic and neurologic factors related to overactive bladder, are known to induce nocturia [39]. Prior studies have also associated obesity, cardiovascular disease, diabetes mellitus, sleep disorders, psychiatric problems and environmental factors with nocturia [40,41]. In addition, endocrine factors related to salt and water imbalances and nocturnal polyuria can influence nocturia [42]. A variety of physical and mental problems are associated with nocturia. Therefore, it was necessary to consider various possible confounders when evaluating the associations between nocturia and falls in the present study.

In this study, falls could have occurred at night upon arousal to void or during the day. To minimize opportunistic falling events that may not have been influenced by any physical or psychological conditions, a further analysis was conducted for falls ≥ 2 instances. This analysis showed that the significant positive relationship between nocturia and falls was maintained in the ≥ 2 falls group (S1 Table).

Our data should be interpreted with caution for several reasons. Although we considered numerous possible factors related to falls, the potential confounding effects of unadjusted variables could not be excluded. Moreover, we could not conduct objective urologic examinations, including the presence of benign prostate hypertrophy and urological metrics to determine urinary flow and residual volume, because of the use of a very large population-based survey. The present study was based on self-reported surveys. Thus, recall bias could have occurred during data acquisition. Nocturia involves various pathophysiological entities, and we intended to assess its relationship with falls. Thus, a validated survey for nocturia might be more suitable and sufficient for the objectives of our present study. The cross-sectional study design limited the value of the correlations between falls and nocturia. Specifically, we could not determine the causal relationship between falls and nocturia. Finally, our study population was of Korean ethnicity. Therefore, the relationship between nocturia and falls could be different in other ethnic groups that might have different physical and environmental characteristics.

The KCHS is a nationwide representative survey using weighted sampling and analysis. Using the KCHS data, this study retrieved more population data than prior studies, which enabled us to adjust numerous confounding factors. Our use of subjects with an identical ethnic background might minimize possible confounding effects from life-style and other unconsidered variables. Furthermore, the KCHS used a standardized questionnaire for nocturia. Overall, this study is the first to demonstrate a dose-dependent relationship between the frequency of nocturia and falls. Future prospective studies of the associations between falling and nocturia will elucidate the direct causality of falls and nocturia.

## Conclusions

The prevalence of falls was approximately 14.6% in adult Korean men. The frequency of nocturia was significantly correlated with falls. The AORs of falls increased as the frequency of nocturia increased. Recurrent arousal from sleep during the night could be directly related to falls and/or could indirectly influence daytime fatigue or other physical and mental health factors that are associated with falls. Further study will be needed to determine causal relationships and the underlying mechanisms between nocturia and falls.

## Supporting Information

S1 Table Adjusted odd ratios of nocturia (0–5 or more instances) for fall down using multiple logistic regression analysis with complex sampling (fall down ≥ 2 instances a year). (DOCX)
