# Supplementary material for: Nocturia Is Associated with Slipping and Falling
Source: PLoS One. 2017 Jan 6;12(1):e0169690. doi: 10.1371/journal.pone.0169690 (PMC5218404; doi:10.1371/journal.pone.0169690)
Supplement: S1 Table — (DOCX) [file pone.0169690.s001.docx]

S1 Table. Adjusted odd ratios of nocturia (0-5 times) for fall down using multiple logistic regression analysis with complex sampling (fall down ≥ 2 times a year).

|  | |  | OR | 95% CI | P-value |
| --- | --- | --- | --- | --- | --- |
| Model 2^b^ | | |  |  | <0.001^a^ |
|  | None | | 1 |  |  |
|  | 1 time | | 1.42 | 1.29-1.56 |  |
|  | 2 times | | 2.21 | 1.93-2.52 |  |
|  | 3 times | | 2.89 | 2.40-3.49 |  |
|  | 4 times | | 3.14 | 2.36-4.18 |  |
|  | ≥ 5 times | | 2.49 | 2.03-3.05 |  |

^a^ Significance at P < 0.05

^b^ Adjusted with age, education, income level, BMI group, smoking, alcohol consumption, stress level, hypertension, diabetes mellitus, cerebral stroke, angina or myocardial infarction, arthritis, and osteoporosis history
